# Role of computed tomography virtual bronchoscopy in a case of pulmonary tuberculosis complicated by bronchopleural fistula and pyopneumothorax

**DOI:** 10.1016/j.radcr.2026.04.019

**Published:** 2026-05-14

**Authors:** Pramesti Indri Miranti, Muhammad Ris Suangkupon Lubis

**Affiliations:** Department of Radiology, Faculty of Medicine, Universitas Padjadjaran, West Java, Indonesia

**Keywords:** Bronchopleural fistula, Pulmonary tuberculosis, Pyopneumothorax, Chest CT-Scan, CT virtual bronchoscopy

## Abstract

Bronchopleural fistula (BPF) refers to an abnormal passage connecting the bronchial tree to the pleural cavity, which may arise from infection, trauma, or post-surgical complications. When associated with tuberculosis (TB), its insidious and slowly progressive nature often delays recognition, complicating timely management. This case highlights a rare presentation of large tuberculous peripheral bronchopleural fistula in a young adult, complicated by massive pyopneumothorax and ultimately requiring pneumonectomy, a rare intervention in this age group. The patient, on his fifth month of anti-tuberculosis therapy, showed symptoms of fever, productive cough, weight loss, and respiratory distress. Imaging revealed left-lung consolidation with “tree-in bud” nodule, and ground-glass opacity, together with extensive right-sided pyopneumothorax. Chest computed tomography and virtual bronchoscopy confirmed a 19-mm fistulous tract arising from the right secondary bronchus. In TB-related BPF, structural lung damage may occur through ruptured cavities or erosion of caseating lymph nodes, forming sinus tracts that permit persistent air and purulent drainage into the pleural space. When conventional bronchoscopy is limited computed tomography and virtual bronchoscopy provide reliable noninvasive visualization of the fistula and airways, enabling accurate diagnosis and management planning.

## Case presentation

A 19-year-old male reported a 4-week history of progressive dyspnea, accompanied by a productive cough, intermittent fever, nocturnal diaphoresis, and significant involuntary weight loss. He had been receiving first-line anti-tuberculosis therapy for the preceding 5 months. One week before admission, he underwent chest tube insertion at another facility for a right-sided hydropneumothorax, which drained approximately 100 mL/day of thick purulent fluid with continuous air bubbling. Despite drainage, his respiratory symptoms failed to improve, prompting referral to our center for further evaluation.

**Fig. 1:**
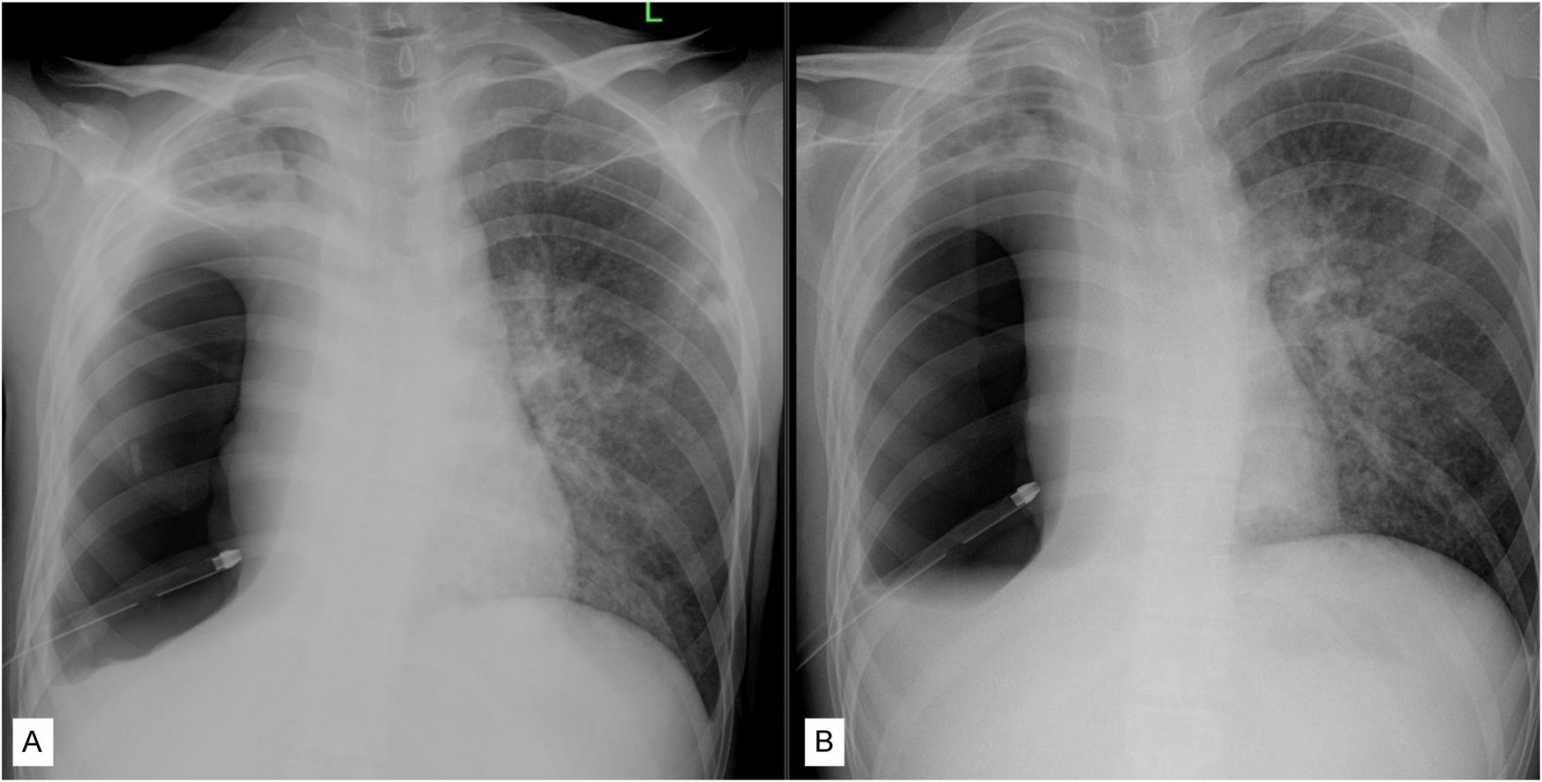
Chest radiography (A) initial chest radiograph at hospital admission showing right-sided pneumothorax, right pleural effusion, and left lung infiltrates; (B) after 7 days hospitalization, showed persistent right pneumothorax.

Upon presentation, the patient appeared acutely ill, tachypneic, and cachectic. Physical examination demonstrated diminished breath sounds over the right hemithorax, with coarse crackles auscultated over the left lung. Laboratory evaluation demonstrated normal leukocyte count, with a history of receiving antibiotics during hospitalization. Human Immunodeficiency Virus status was not evaluated. At the time of admission, chest radiography revealed a right-sided pneumothorax, right pleural effusion, and infiltrates in the left lung field. A chest tube thoracostomy (CTT) had already been inserted at the time of admission; however, after 7 days in our hospital, there was no radiologic improvement in either the pneumothorax or the pleural effusion (Fig. 1). Analysis of the pleural fluid demonstrated elevated protein (4140 mg/dL) and markedly increased LDH (9771 U/L), indicating empyema.

**Fig. 2:**
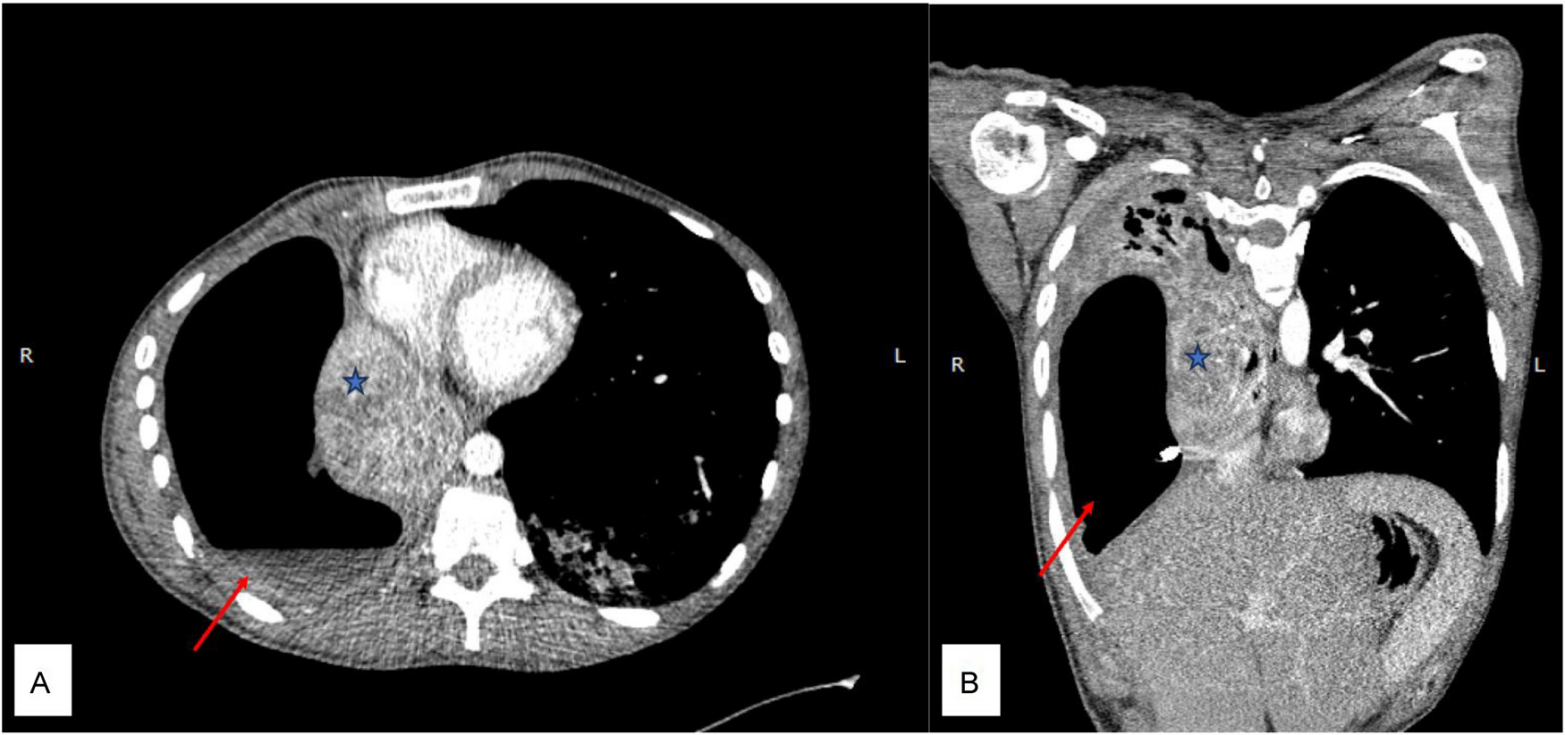
Chest computed tomography with contrast mediastinum window with axial (A) and coronal view (B), indicating large right pyopneumothorax (red arrow), right lobe collapse (blue star) and right lobe cavity continuing into the pleural area, suggesting a bronchopleural fistula.

**Fig. 3:**
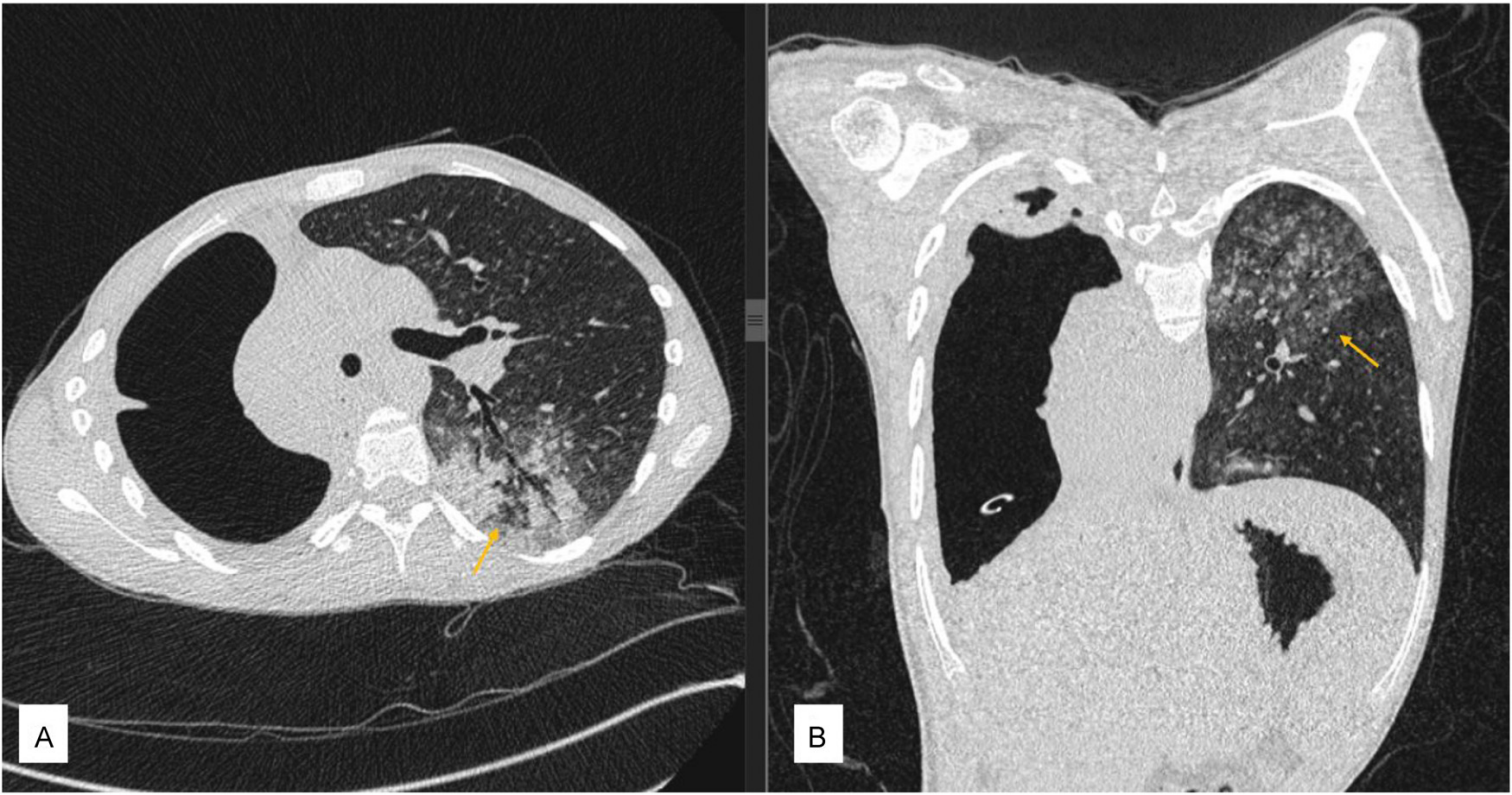
Chest computed tomography lung window axial view (A) and coronal view (B) revealed centrilobular nodules “tree-in-bud” surrounded with ground-glass opacity and patchy consolidation in the left lung (orange arrow).

A contrast-enhanced chest computed tomography (CT) scan showed a large right-sided pyopneumothorax with atelectasis of the right lung parenchyma. The lung collapsed appeared to extend into the pleural cavity, strongly suggesting a peripheral bronchopleural fistula (Fig. 2), although this finding was further supported by virtual bronchoscopy, with mediastinal shift and herniated left lung to the right sided. Multiple centrilobular nodules with a “tree-in-bud” pattern surrounded by ground-glass opacities and patchy consolidation were noted in the left lung, suggestive of a typical radiographic appearance of tuberculosis (Fig. 3).

**Fig. 4:**
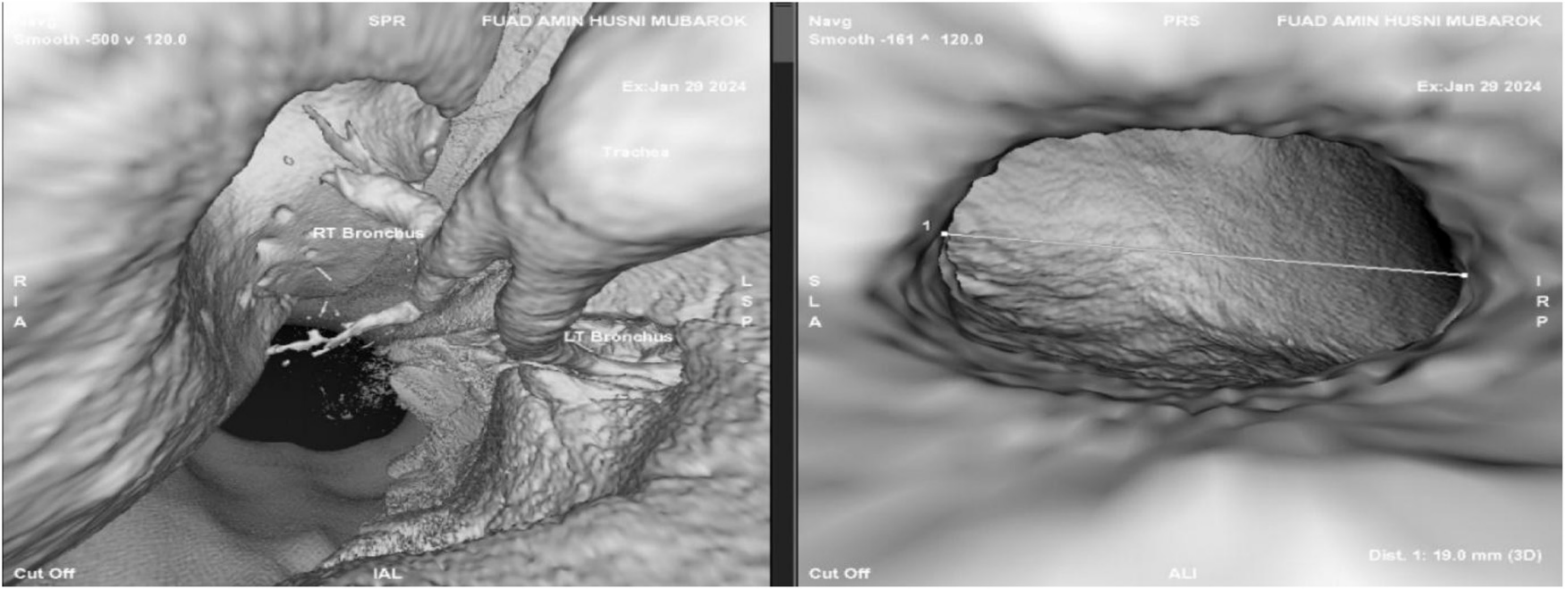
CT virtual bronchoscopy revealed a fistulous tract in the right secondary bronchus measuring 19 mm.

Virtual bronchoscopy examination revealed a bronchopleural fistula connecting the right secondary bronchus to the pleural space, with an estimated diameter of 19 mm (Fig. 4). Given the extensive lung destruction and persistent pleural contamination, surgical intervention was deemed necessary. The patient underwent a thoracotomy followed by right pneumonectomy. During the procedure, approximately 1000 cc of purulent material was identified, and the right lung was found to be necrotic.

## Discussion

Pulmonary tuberculosis (TB) continues to pose a major global public health challenge, affecting millions of individuals annually. Patients with TB commonly exhibit symptoms such as a productive cough, dyspnea, fever, unintentional weight loss, night sweats, and hemoptysis. The clinical presentation in patients with bronchopleural fistula (BPF) varies according to the size of the fistula and the chronicity of the infectious process [1,2]. Notably, the productive cough often becomes more pronounced when the patient assumes a lateral decubitus position with the unaffected lung dependent, opposite to the involved hemithorax [3]. In this case, the patient reported a 4-week history of dyspnea accompanied by productive cough, weight loss, intermittent fever, and night sweats.

Cavitations caused by *Mycobacterium tuberculosis* within the airways may rupture, allowing dissemination of infectious material into the pleural cavity and triggering progressive pleural inflammation and tissue injury. Ongoing chronic inflammation can result in fibrotic remodelling and the development of sinus tracts connecting the bronchial tree to the pleural space, known as bronchopleural fistula (BPF). Sustained air leakage may give rise to pneumothorax and empyema, potentially progressing to pyopneumothorax. Tuberculous BPF represents a rare complication, typically arising from longstanding cavitary pulmonary disease or erosion of tuberculous lymph nodes, culminating in pathological communication between the bronchial system and the pleural cavity [1,4,5].

The clinical manifestations of bronchopleural fistula (BPF) are variable and may include a productive cough, subcutaneous emphysema, fever, acute-onset dyspnea, hypoxemia, hypotension, and the development of pneumothorax. Additionally, a characteristic indication of this complication is the rapid accumulation of fluid in the water-seal drainage over the first few days of management, accompanied by signs of pleural leakage such as continuous bubbling within the drainage system [3]. Recurrent or persistent pleural effusion, especially after medical procedures, may indicate the presence of a bronchopleural fistula [[5], [6], [7]]. In this patient, the continuous drainage of fluid accompanied by air bubbling that further raised the suspicion of an underlying bronchopleural fistula.

A bronchopleural fistula is defined as a pathological communication between the bronchial tree and the pleural cavity, which may develop secondary to pulmonary malignancies, pneumonia, empyema, blunt or penetrating thoracic trauma, or as a complication following thoracic surgical procedures. Recent cases highlighted show that BPF is more often caused by tuberculosis than by surgery. Consistently, the present study also demonstrated that the majority of BPF cases occurred in patients with tuberculosis, and TB was significantly associated with a higher incidence of secondary attack BPF prior to surgery, indicating a greater predisposition of TB patients to develop recurrent or persistent bronchopleural fistula [8,9]. In this patient previously diagnosed with tuberculosis, presented with a persistent pleural effusion that failed to improve despite chest tube drainage, thereby raising concern for an underlying bronchopleural fistula.

The diagnosis of bronchopleural fistula (BPF) involves assessing clinical symptoms, imaging findings indicating air leakage, and bronchoscopic evaluation. Contrast-enhanced chest computed tomography is highly effective for outlining fistulous pathways and determining their size. However, bronchoscopy remains the preferred diagnostic method, providing direct visualization and allowing procedures like sequential balloon occlusion for accurate localization. Additional diagnostic tools include planar imaging, single-photon emission computed tomography (SPECT), and computed tomography bronchography (CTB), with CTB being especially beneficial for detailed visualization of the fistula tract [7,10,11]. CT typically demonstrates the presence of air and fluid within the pleural cavity, with or without visualization of a direct tract or communication between the airway or lung parenchyma and the pleural space. But may present as localized regions of lung consolidation with low attenuation that appear to connect with an adjacent empyema, or as distinct interruptions in the continuity of the visceral pleura [12]. Rare cases of tuberculous bronchopleural fistula and emphasized that the fistulous tract is more readily identified using thin-section multidetector computed tomography (MDCT) with multiplanar reformations (MPR) and 3-dimensional (3D) reconstruction. Similarly, there was a case of bronchopleural fistula in a patient with necrotizing pneumonia due to *Mycobacterium avium* complex, with the diagnosis confirmed by contrast-enhanced chest computed tomography [13]. Chest CT scans play a pivotal role in accurately delineating the location, morphological characteristics, and size of a BPF. In this case, the patient underwent a chest CT scan with contrast and revealed pyopneumothorax and BPF of the right lung.

CT-based virtual bronchoscopy provides a noninvasive, 3-dimensional reconstruction of the tracheobronchial tree, enabling simulated intraluminal visualization derived from helical CT images. This modality is particularly valuable for assessing airway abnormalities when conventional bronchoscopy is contraindicated or technically limited, as it allows evaluation of the airway lumen up to the fourth-order bronchi and offers a complete view beyond sites of obstruction. Its detailed mapping of airway caliber and angulation supports pre-procedural planning, such as bronchial stent placement, and facilitates assessment of potential complications after stent deployment. Because it avoids instrumentation of the airway, virtual bronchoscopy can be safely repeated to monitor post-treatment progress, evaluate bronchial stump dimensions, and assist in determining suitability for sealants. When combined with other imaging modalities, it provides complementary information about the precise location and extent of a bronchopleural fistula, thereby aiding therapeutic decision-making. However, its diagnostic accuracy may be limited by airway occlusion, patient motion, unlike conventional bronchoscopy it cannot be used for therapeutic interventions [[14], [15], [16]]. In the case presented, CT-based virtual bronchoscopy was employed and successfully identified a bronchopleural fistula in the right secondary bronchus with a diameter of 19 mm. Following diagnostic confirmation, the patient was subjected to thoracotomy with surgical resection of the right lung.

## Conclusion

Although uncommon, tuberculous bronchopleural fistula presents significant diagnostic and therapeutic difficulties. Non-invasive imaging modalities such as chest CT and virtual bronchoscopy provide valuable information regarding the location and extent of the fistulous tract. These techniques can augment clinical evaluation and support timely surgical decision-making, particularly in situations where standard bronchoscopy cannot be performed.

## Patient consent

Complete written informed consent was obtained from the patient for the publication of this study and accompanying images.
